# The Effect of Traditional Aerobic Exercise and Sprint Interval Training on Insulin Resistance in Men With Prediabetes: A Randomised Controlled Trial

**DOI:** 10.7759/cureus.20789

**Published:** 2021-12-29

**Authors:** Khaled M Badaam, Urjita S Zingade

**Affiliations:** 1 Physiology, Government Medical College and Hospital, Aurangabad, Aurangabad, IND; 2 Physiology, Rajarshee Chhatrapati Shahu Maharaj Government Medical College, Kolhapur, IND

**Keywords:** pre-diabetes, body mass index (bmi ), vigorous exercise, endurance exercise, homeostatic model assessment of insulin resistance (homa-ir), high intensity exercise

## Abstract

Introduction

Prediabetes is an intermediate stage with hyperglycaemia below the threshold of diabetes mellitus. Insulin resistance is a significant factor in its pathogenesis. Lifestyle modifications are suggested and found to be more beneficial in this stage. Moderate-intensity exercise for 30 to 45 minutes a day is routinely recommended but has low compliance, and lack of time is a significant deterrent. Sprint interval training (SIT) is an alternate exercise regimen with higher intensity and less time requirement. The present study compares the effect of a three-month intervention of traditional aerobic exercise and sprint interval training on insulin resistance in prediabetic men.

Methods

The study subjects were males aged 25 to 40 years with prediabetes as per the American Diabetes Association criteria of fasting and two-hour plasma glucose levels. The study is a parallel-group randomised trial with one arm (AE group) involved in the traditional aerobic exercise (brisk walking) for 30 minutes, five days a week. The other arm was the sprint interval training (SIT) group performing an ‘all-out’ run effort for one minute followed by a recovery rest period of one and a half minutes, completing one cycle of two and half minutes. Four such cycles were completed in each session. Thus, the exercise sessions were just 10 minutes daily, three days a week. The duration of the intervention was three months. One hundred and sixty participants were recruited after screening and randomly assigned in a 1:1 ratio to the two groups. The primary outcome measure was insulin resistance estimated by homeostasis model assessment -estimated insulin resistance (HOMA-IR). The secondary outcome measures were fasting plasma glucose and serum insulin, glycated haemoglobin, body mass index and waist-hip ratio.

Results

The mean age of the AE group was 30.7 ± 3.3 years, and the SIT group was 31 ± 3.4 years. Seventy-two men from the AE group and 74 from the SIT group completed the study. After the three-month AE and SIT exercise, the per-protocol analysis reflected a significant reduction in insulin resistance, i.e., HOMA-IR (3.6 ± 1.1 to 3 ± 1.2, p<0.0001) after traditional aerobic exercise. Similarly, the HOMA-IR was significantly reduced after sprint interval training (3.3 ± 1.2 to 2.5 ± 1, p<0.0001). The intention-to-treat analysis also found that the reductions in HOMA-IR after both exercise protocols were statistically significant. The change in insulin resistance compared for the SIT vs AE group was not statistically significant. Secondary outcome measures HbA1c, fasting glucose, fasting insulin, BMI, and waist-hip ratio showed significant improvement with AE and SIT.

Conclusions

The sprint interval training similarly improved insulin resistance and other parameters compared to the traditional exercise group. SIT can be a time-efficient exercise protocol suggested as a part of lifestyle modification for men with prediabetes.

## Introduction

Diabetes and prediabetes prevalence is rising rapidly in India due to the combination of genetic predisposition and lifestyle changes [1]. Prediabetes is an intermediate hyperglycemia stage with an increased risk of cardiovascular disease. Insulin resistance and hyperinsulinemia are significant contributing factors for the increased predisposition towards cardiovascular disease [2-5]. Exercise is a significant component of lifestyle modification suggested for the management of prediabetes and prevention/delay in the progression towards diabetes. The exercise recommendations of 150 minutes per week at moderate-intensity, although beneficial, have not resulted in high compliance. Lack of time is the frequent reason for non-adherence [6-8]. It has been reported that prediabetes individuals are more likely to benefit from exercise. However, low- to moderate-intensity exercise is not found to reverse altered glucose homeostasis in prediabetes completely, and it is recommended to explore exercise at different intensities [9]. Thus, there is a need for higher intensity exercise regimens with less time commitment for better application in prediabetes management. Sprint Interval Training (SIT) has gained interest as a high-intensity exercise with a shorter time commitment. Recent studies have reported that SIT is a time-efficient and effective exercise model for improving insulin sensitivity and other metabolic and vascular health parameters [10-12]. The present study compares three months of sprint interval training with traditional aerobic exercise for changes in insulin resistance in prediabetic men.

## Materials and methods

The study design is a parallel-group randomized controlled trial comparing the effects of traditional aerobic exercise and sprint interval training on insulin resistance in prediabetes. The trial was conducted at a government medical institute in Maharashtra, India. The sample size was calculated as 73 subjects per group for two-tailed hypothesis testing (α set as 5% and the power of study as 80%) to compare the change in insulin resistance (homeostasis model assessment - estimated insulin resistance [HOMA-IR]) between the two exercise groups based on the assumed difference in HOMA-IR of 25%, moderate effect size (Cohen's d). The sample size enrolled was 80 in each group, taking into account the possible loss to follow-up. The ethics committee of the institute approved the study protocol (Institutional Ethics Committee - Government Medical College Aurangabad vide letter: Pharma/IEC-GMCA/477/2011). All the study subjects gave informed written consent in their known language. Trial registration with the Clinical Trial Registry of India (CTRI/2012/09/002974) and the World Health Organization registry network (URN: U1111-1133-9202) was done. Helsinki declaration principles were followed while conducting the trial.

Participants and eligibility criteria

Eligible participants were adults aged 25 to 40 years with prediabetes, asymptomatic males with normal baseline electrocardiogram (ECG), and willing to participate in the study. Prediabetes screening was done as per the fasting plasma glucose (100 to 125 mg/dL) or 2-hour glucose (140 to 199 mg/dL) as defined by American Diabetes Association (ADA) [7]. All known cases of cardiac disease, lung disease, renal disease, hepatic disease, diabetes, or any disease with contraindication to exercise, prediabetes patients on pharmacotherapy, smokers, blood pressure above 160/100 mmHg, those already doing regular exercise or sports, those not available for intervention period were excluded. Participants were recruited from the Government Medical College, private hospitals, medical camps and direct contacts.

Outcome measures

The primary outcome measure was insulin resistance estimated by HOMA-IR, as Matthews et al. described [13]. Secondary outcome measures reported: fasting glucose and fasting insulin, glycated haemoglobin (HbA1c), body mass index (BMI), and waist-hip ratio (WHR).

Screening

History taking, physical examination, ECG, fasting glucose, 2-hour glucose were performed in the subjects. Height measurement was done by a wall-mounted stadiometer. An electronic weighing machine (precision, 0.1 kg) measured body weight in light clothing before breakfast. BMI was calculated as weight in kg/(height in m)2. Waist circumference at the midpoint between the anterior superior iliac crest and the lowest rib and the hip circumference at the level of the maximal gluteal protrusion was measured. The WHR was calculated as waist circumference in cm/hip circumference in cm [14]. Screening of 237 high-risk subjects was done based on ADA reports (BMI > 25 kg/m2, first degree relative of diabetic, physical inactivity) [15,16]. Seventy-seven subjects were excluded as 39 were normoglycemic, seven were having diabetes, nine subjects had high blood pressure, 13 declined for the 3-month exercise protocol, and the physician excluded nine subjects. The enrolled 160 subjects were allotted serial numbers.

Sample collection and its evaluation

After a 12-hour overnight fast, 5 mL of venous blood was collected in a syringe to measure HbA1c, plasma glucose, and serum insulin. Plasma glucose (mg/dL) estimation was done by the glucose oxidase peroxidase (GOD-POD) method [17]. Serum insulin (µU/mL) estimation was done by electrochemiluminescence immunoassay (ECLIA®; Elecsys, Roche, Mannheim, Germany). HbA1c (%) measurement was done using ion-exchange high-performance liquid chromatography (HPLC) (D-10™, Bio-Rad, Hercules, CA, USA). HOMA-IR was calculated according to the following formula: homeostatic model assessment (HOMA-IR) = (fasting insulin in micro-IU/mL * fasting glucose in mg/dl)/405 (based on Matthews et al. [13]).

Randomization and intervention

Eligible participants were randomly assigned centrally by a computer-generated random-number sequence in a 1:1 ratio to either traditional aerobic exercise (AE) group or Sprint Interval Training (SIT) group. Random assignments were done in 10 waves of 16, with equal randomization in each wave. Allocation concealment was done by opaque numbered sealed envelopes. The supervising trainers were separated into two groups, and the participants and trainers did not know about the other group. 

The AE group exercised at moderate intensity (brisk walking) for 30 minutes a day, five days a week. The SIT group exercised with an ‘all-out’ run effort for one minute followed by a recovery rest period of one and a half minutes, completing one cycle of two and half minutes. Four such cycles were completed in each session. Thus, the exercise sessions were just 10 minutes a day. Warm-up and stretching exercises were done before and after the sessions for 5 minutes. There were three sessions per week with at least one-day gap between two consecutive sessions. The weekly time spent on exercise was 30 minutes for SIT (additional 15 minutes for warm-up) compared to 150 minutes per week for the traditional aerobic exercise. This intervention was field-based and in real-life conditions. Participants were asked to maintain their usual dietary habits and physical activity levels. Compliance with >80% sessions was considered in the analysis. The intervention period was three months (target of 36 sessions of SIT and 60 sessions of AE). After the intervention, participants underwent repeat evaluation of the outcome parameters between 48 to 72 hours after the last exercise session. Out of the 160 subjects, 146 subjects were included for the post-intervention testing. An independent doctor conducted and supervised the before and after evaluation and testing of the participants. The outcome assessors, those involved in investigations and the statistician were blinded regarding allocation.

Statistical analysis

The analysis was performed using Microsoft Excel 2017 (Microsoft Corporation, Redmond, USA) and online statistical calculators available at https://www.socscistatistics.com and http://www.graphpad.com/quickcalcs. Primary outcome variable HOMA-IR was assessed as per per-protocol analysis (n = 74 + 72 = 146), and the intention-to-treat (ITT) analysis (n = 80 + 80 = 160) with the baseline observations carried forward (BOCF). A test for the normal distribution of data (Kolmogorov-Smirnov test) was used. Parametric (paired and unpaired t-test) tests were used for normally distributed data; otherwise, nonparametric tests (Wilcoxon signed-rank test and Mann-Whitney U test) were applied. For within-group comparison, paired t-test or Wilcoxon signed-rank test was used, and for between-group comparison, unpaired t-test or Mann-Whitney U test was used. 

## Results

**Figure 1:**
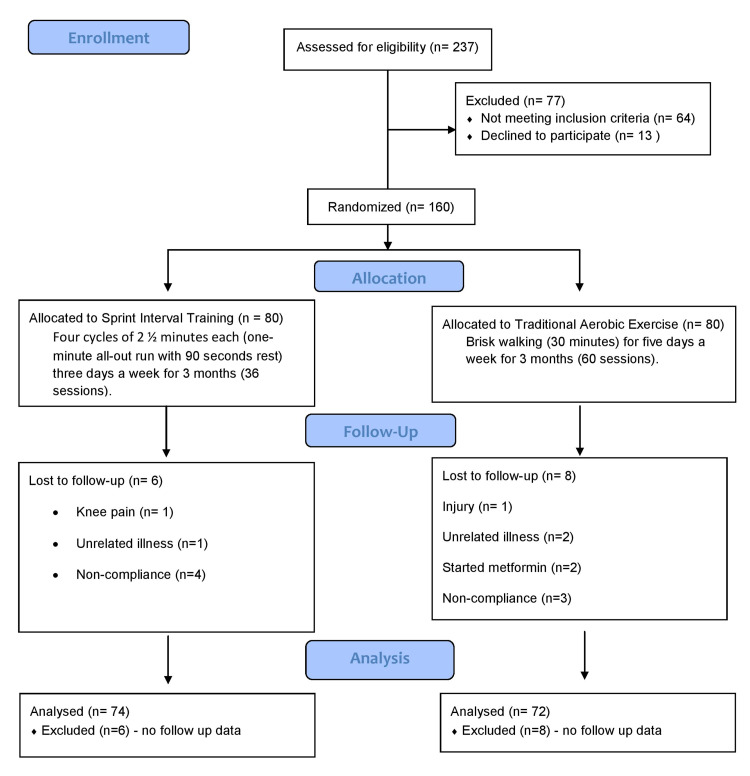
Flow diagram of the study

One hundred forty-six subjects completed the exercise protocols and the post-intervention evaluation; 72 were from the AE group and 74 from the SIT group. Figure 1 depicts the study flow chart.

The mean age of the subjects of the AE group was 30.7 ± 3.3 years, and the SIT group was 31 ± 3.4 years. Baseline characteristics of the study subjects in both groups were comparable, as shown in Table 1. 

**Table 1:** Baseline characteristics of the study subjects AE: Traditional aerobic exercise, SIT: Sprint Interval Training, SD: Standard Deviation

Variables	AE group (n=80) (Mean ± SD)	SIT group (n=80) (Mean ± SD)
Age (Years)	30.7 ± 3.3	31 ± 3.4
Height (meters)	1.73 ± 0.03	1.73 ± 0.04
Weight (kg)	84.2 ± 7.6	85.9 ± 8.3
BMI (kg/m^2^)	28.2 ± 2.9	28.6 ± 3.1

After the three-month AE and SIT exercise, the per-protocol analysis reflected a significant reduction in insulin resistance, i.e. HOMA-IR (3.6 ± 1.1 to 3 ± 1.2, p<0.0001) after traditional aerobic exercise. Similarly, the HOMA-IR was significantly reduced after sprint interval training (3.3 ± 1.2 to 2.5 ± 1, p<0.0001) [Table 2]. 

**Table 2:** Per-protocol analysis of insulin resistance (HOMA-IR) HOMA-IR: Homeostatic model assessment - Insulin Resistance, AE: Traditional aerobic exercise, SIT: Sprint Interval Training, SD: Standard Deviation, CI: Confidence Interval, **statistically highly significant

Variable	AE Group (n=72) (Mean ± SD)				SIT Group (n=74) (Mean ± SD)			
	Pre-AE	Post-AE	Mean Difference [95% CI]	p-value	Pre-SIT	Post-SIT	Mean Difference [95% CI]	p-value
HOMA-IR	3.6 ± 1.1	3 ± 1.2	-0.6 [ -0.87 to -0.34]	<0.0001^**^	3.3 ± 1.2	2.5 ± 1	-0.8 [-1.12 to - 0.39]	<0.0001^**^

The intention-to-treat analysis also found that the reductions in HOMA-IR after both exercise protocols were statistically significant [Table 3]. 

**Table 3:** Intention to Treat analysis of Insulin resistance (HOMA-IR) HOMA-IR: Homeostatic model assessment - Insulin Resistance, AE: Traditional aerobic exercise, SIT: Sprint Interval Training, SD: Standard Deviation, CI: Confidence Interval, **statistically highly significant

Variable	AE Group (n=80) (Mean ± SD)				SIT Group (n=80) (Mean ± SD)			
	Pre-AE	Post-AE	Mean Difference [95% CI]	p-value	Pre-SIT	Post-SIT	Mean Difference [95% CI]	p-value
HOMA-IR	3.6 ± 1.1	3.1 ± 1.2	-0.55 [ -0.78 to -0.31]	<0.0001^**^	3.3 ± 1.2	2.6 ± 1	-0.7 [-1.04 to - 0.36]	<0.0001^**^

The change in insulin resistance compared for the SIT vs AE group was not statistically significant [Table 4]. 

**Table 4:** Comparison of change observed in Insulin resistance (HOMA-IR) between groups HOMA-IR: Homeostatic model assessment - Insulin Resistance, AE: Traditional aerobic exercise, SIT: Sprint Interval Training, SD: Standard Deviation, CI: Confidence Interval

Variable	Per Protocol Analysis				Intention to Treat Analysis			
	Change in AE Group (Mean ± SD)	Change in SIT Group (Mean ± SD)	Mean Difference [95% CI]	p-value	Change in AE Group (Mean ± SD)	Change in SIT Group (Mean ± SD)	Mean Difference [95% CI]	p-value
HOMA-IR	-0.6 ± 1.1	-0.8 ± 1.6	0.2 [-0.274 to 0.620]	0.445 (NS)	-0.5 ± 1.1	-0.7 ± 1.5	0.2 [-0.237 to 0.587]	0.403 (NS)

Secondary outcome measures HbA1c, fasting glucose, fasting insulin, and BMI showed significant improvement with AE and SIT. However, there was no significant difference between the groups regarding improving these parameters [Table 5]. There was a significantly more decrease in WHR in the SIT group, but the difference in means was approximately 1% only [Table 5]. Among the SIT group subjects, one subject could not complete the intervention due to knee pain. The pain subsided over a week; however, the subject did not continue with the study. There was a sprain injury in the AE group, and the subject withdrew from the study. There were no other adverse events.

**Table 5:** Comparison of secondary outcome variables before and after AE and SIT NS: statistically not significant,  * statistically significant, **statistically highly significant; AE: Traditional aerobic exercise, SIT: Sprint Interval Training, SD: Standard Deviation, BMI: Body mass index, HbA1c: glycated haemoglobin, WHR: waist-hip ratio

Variables (Mean ± SD)		Pre-AE	Post-AE	p-value	Pre-SIT	Post-SIT	p-value	Difference (AE)	Difference (SIT)	p-value
BMI		28.2 ± 2.8	27.6 ± 2.7	<0.0001**	28.7 ± 3.2	28.3 ± 2.9	<0.0001**	-0.55 ± 0.58	-0.45 ± 0.59	0.32 (NS)
HbA1c (%)		5.9 ± 0.3	5.6 ±0.3	<0.0001**	5.9 ± 0.3	5.5 ± 0.3	<0.0001**	-0.3± 0.3	-0.4 ± 0.3	0.064 (NS)
Glucose (mg/dl)	0 min	107.1 ± 12	102.3 ± 13.9	0.0006**	108.4 ± 11.1	102.8 ± 11.1	<0.0001**	-4.8 ± 11.5	-5.6 ± 10.9	0.670 (NS)
Insulin (µU/ml)	0 min	13.8 ± 4.2	11.8 ± 3.9	<0.0001**	12.3 ± 4.4	10.1 ± 3.8	0.001*	-1.9 ± 4	-2.3 ± 5.9	0.683 (NS)
WHR		0.95 ± 0.04	0.94 ± 0.04	<0.0001**	0.96 ± 0.04	0.95 ± 0.04	<0.0001**	-0.01 ± 0.01	-0.02 ± 0.01	0.001*

## Discussion

Sprint training on the field with just four all-out runs of one minute in each session for three sessions per week for three months showed significant improvement in insulin resistance (HOMA-IR). The benefit was similar to the traditional aerobic exercise of 150 minutes a week. The improvement in insulin resistance was slightly better in SIT group, although the difference was not statistically significant. The decrease in insulin resistance was reflected in improved glycated haemoglobin levels after exercise intervention. The body mass index and waist-hip ratio improvement were observed in both groups. Intermittent exercise at higher intensity stimulates glucose disposal; the molecular level explanation has been given by a higher translocation of glucose transporter type 4 (GLUT4), depletion of glycogen after exercise sessions, and vasodilation [18]. There is an activation of larger muscle mass in sprint interval training compared to moderate-intensity exercise, which is associated with a very high turnover of glycogen breakdown. There is rapid glycogen degradation due to metabolic stress during high-intensity contractions. Thus, after sprint exercise, greater muscle mass will require replenishing the glycogen stores compared to moderate-intensity exercise. This remodelling of the glycogen pool has been described to be an essential factor in regulating insulin sensitivity [11, 19]. Ryan BJ et al. recently compared moderate-intensity exercise with short duration high-intensity exercise. The muscle glycogen depletion, which is an essential contributor to an increase in insulin sensitivity, was found to be similar after just 10 minutes of high-intensity exercise compared with 45 minutes of moderate-intensity exercise. They mentioned that the findings were significant, given that lack of time is a frequent reason for low adherence to exercise guidelines [8, 20]. Our study results are in line with growing literature on the subject.

The recent 2018 Physical Activity Guidelines Advisory Committee report has reviewed the impact of high-intensity training on cardiometabolic risk profile and found it to have benefits similar to moderate-intensity exercise. The report supported the advocation of higher intensity exercise regimens for improving insulin sensitivity, body composition and blood pressure, especially in those at risk of cardiovascular disease and diabetes. The report stressed the need for continued research on the subject, especially longer than six-month duration studies. There is a need for more extended duration studies to assess adherence and benefit on cardiometabolic parameters in the long term [21]. Weston KS et al. reviewed the literature on the subject. They reported that 12-week high-intensity interval training is better than moderate-intensity exercise in improving insulin sensitivity and glucose homeostasis. It was reported as a safe exercise modality with improved quality of life [22]. Gillen JB et al. reported that just 3 minutes of sprint interval training within a total 30-minute time per week is as effective as the weekly 150 minutes exercise in the sedentary population for improving insulin sensitivity and muscle mitochondrial content [23]. High-intensity exercise has higher adherence than moderate-intensity continuous exercise in prediabetes subjects [24]. 

Limitations of the study are the inclusion of males only and the assessment of only fasting status insulin sensitivity. The dietary and other factors were not recorded. The ‘all-out’ effort is subjective and may underestimate the effect if the intensity was not achieved. The study did not have a control group with no exercise prescription to give a better perspective on the exercise effects. Further studies from multiple centres and over a longer duration are needed to establish the role of sprint interval training in managing prediabetes.

## Conclusions

Sprint interval training improved insulin resistance, glycaemic indices and anthropometric parameters similar to traditional aerobic exercise. Considering the significantly less time requirement, this can be an excellent option for people with time constraints. With due precautions, men with prediabetes can be recommended sprint interval training as a time-efficient exercise intervention that can alter the progression towards diabetes. The improvement in insulin resistance in both the exercise groups signifies the benefit of physical activity in prediabetes. There is a need to properly communicate the exercise duration and intensity options to the general public. More public awareness regarding exercise is the need of the hour to tackle the rapid rise in diabetes prevalence.
